# Supplementary material for: Ectopic Expression of PtrLBD39 Retarded Primary and Secondary Growth in Populus trichocarpa
Source: Int J Mol Sci. 2024 Feb 12;25(4):2205. doi: 10.3390/ijms25042205 (PMC10889148; doi:10.3390/ijms25042205)
Supplement: Supplementary file 1 [file ijms-25-02205-s001.zip › Figures S1-S8.pdf]

Ectopic expression of *PtrLBD39* retarded primary and secondary growth in *Populus trichocarpa*

Supplementary Figures

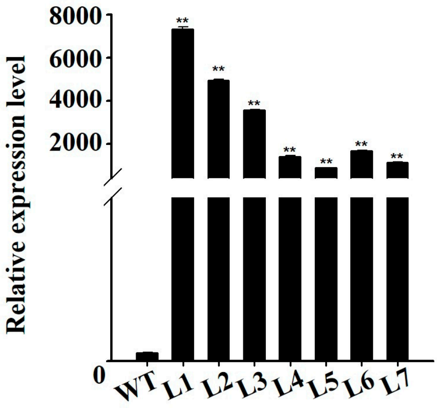

Figure S1. Expression levels of the *PtrLBD39* in stem-xylem tissue of *OE-PtrLBD39* transgenic plants. The error bars represent SE (standard error) values from three biological replicates. Asterisks indicate significant differences between each line of the transgenics and wild-type (WT) plants by Student's *t*-test (** $P < 0.01$)

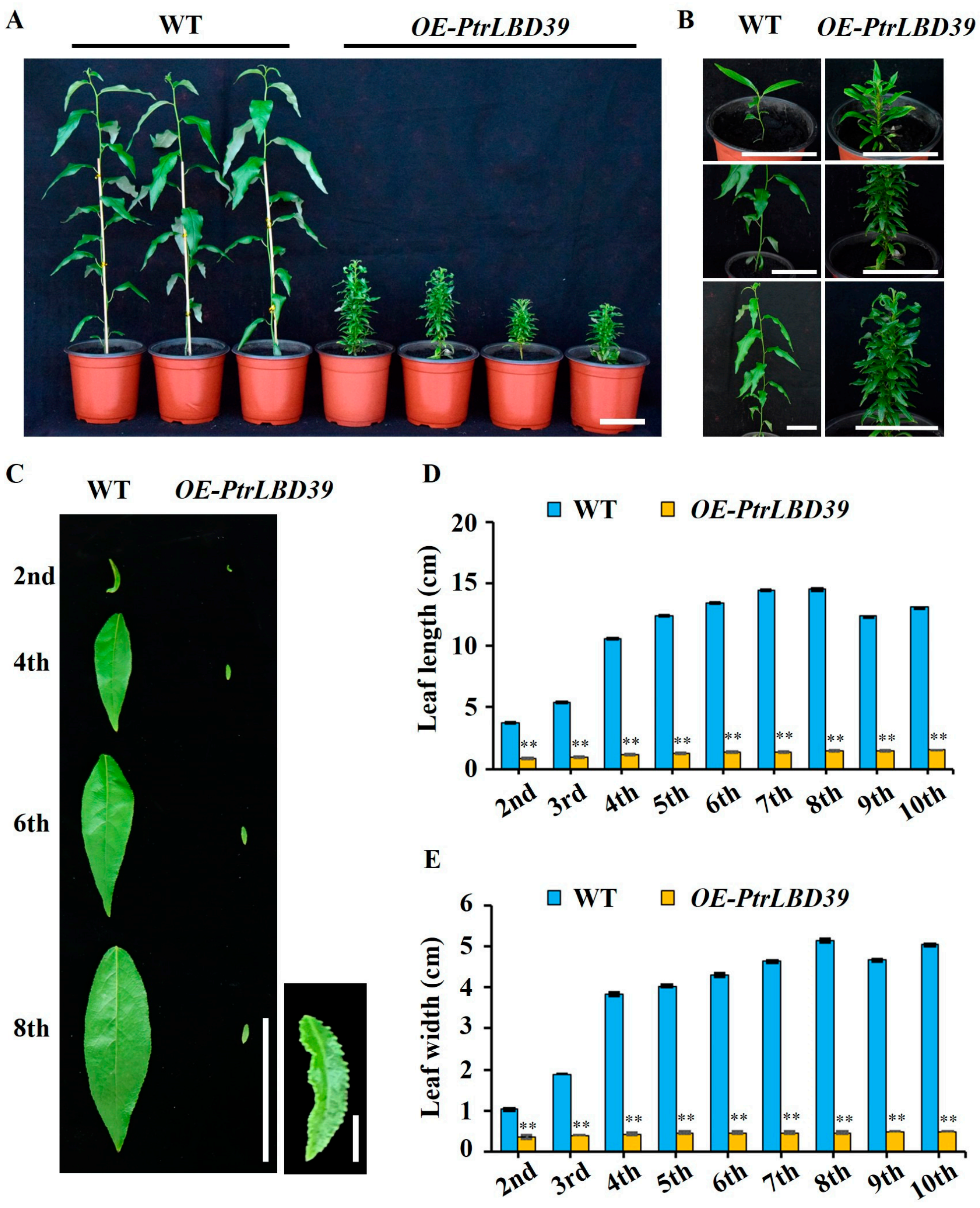

Figure S2. Morphology of *OE-PtrLBD39* transgenic plants. (**A**) *P. trichocarpa* wild-type and *PtrLBD39* overexpression plants at 120 days. (**B**) Leaf morphology observations of wild-type and *PtrLBD39* overexpression plants at 30 days, 60 days and 90 days from top to bottom respectively. Bars = 10 cm. (**C**) Leaf morphology observations of wild-type and *PtrLBD39* overexpression plants at 120 days. Bar = 10 cm. The image on the right shows a magnification of an *OE-PtrLBD39* leaf, Bar = 0.5 cm. (**D and E**) Statistics of length and width of leaves at different stem nodes of wild type and transgenic plants. Error bars represent SE values of three independent experiments. Asterisks indicate significant differences between the transgenics and wild-type plants by Student's *t* test (** $P < 0.01$).

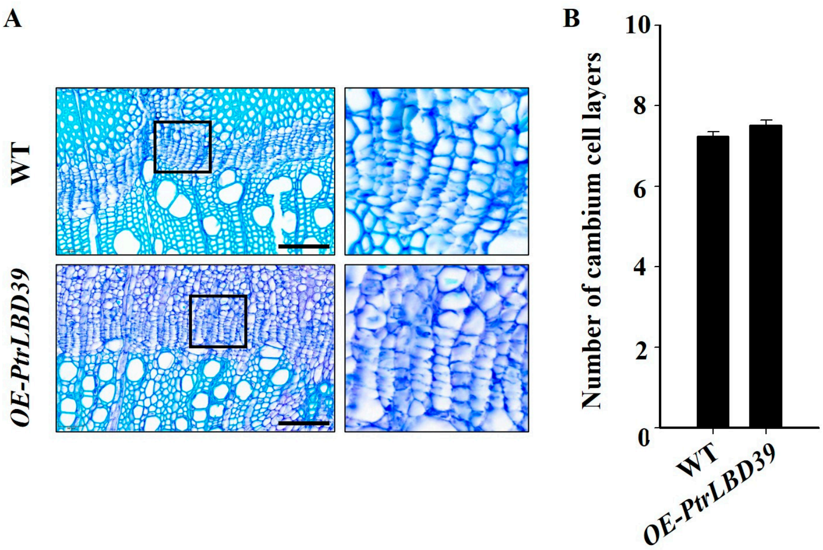

Figure S3. Morphological observation of cambium cells in WT and *OE-PtrLBD39*-L1. (**A**) The 20th internode of *OE-PtrLBD39*-L1 and WT toluidine blue staining observation (Bars =100 μm). (**B**) Number of cambium cell layers in stem vascular tissues of the WT and *OE-LBD3*9-L1 transgenics. The error bars represent SE values from three biological replicates.

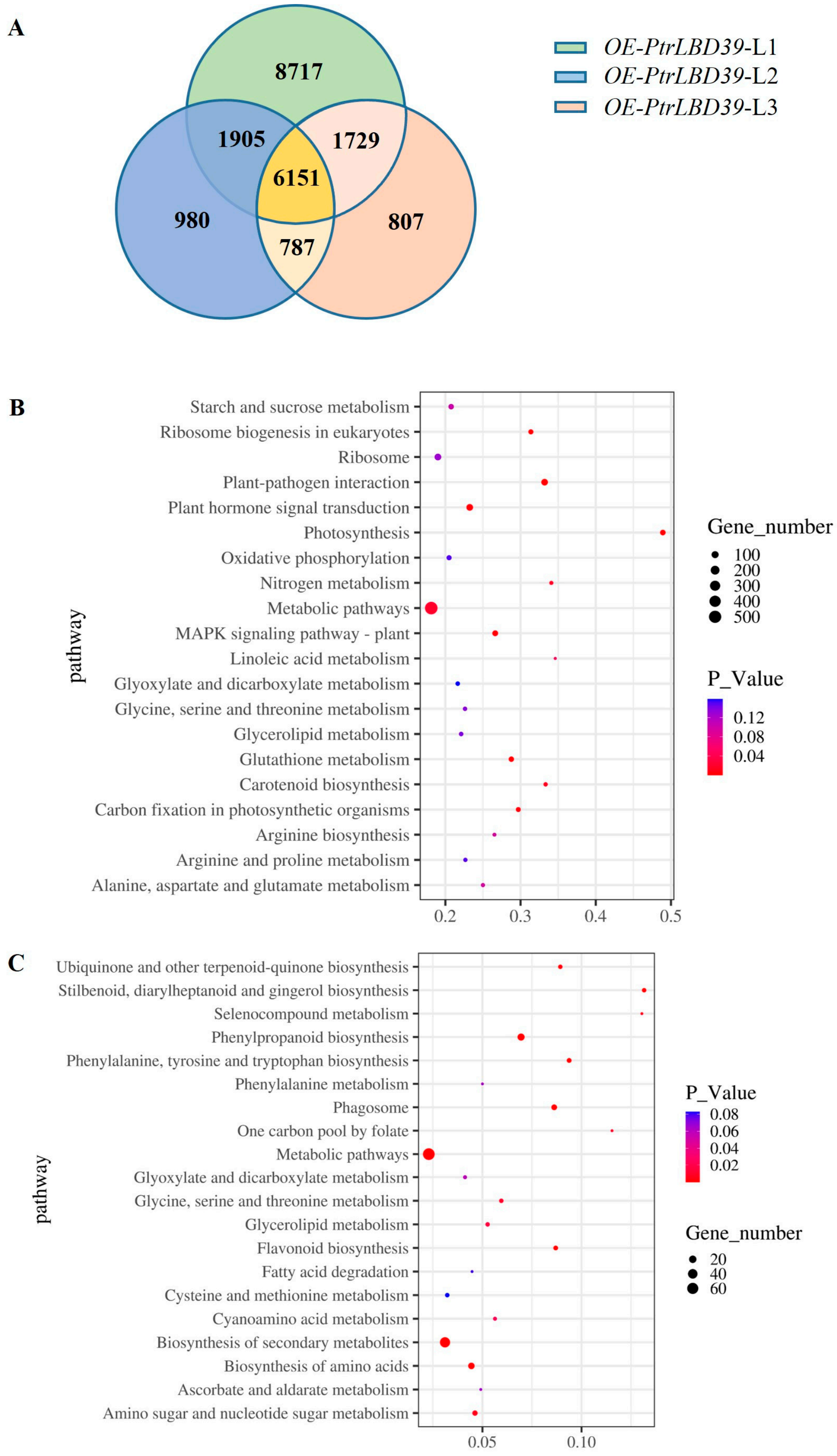

Figure S4. Venn diagram and pathway functional enrichment analysis of DEGs in stem-xylem of *OE-PtrLBD39* transgenic plants. (**A**) Venn diagram showing the common genes between RNA-seq DEGs (FDR < 0.05, |LogFC| > 1) of *OE-PtrLBD39* transgenics. (**B and C**) Pathway functional enrichment analysis of differentially expressed genes co-upregulated (**B**) and down-regulated (**C**) in three lines of *OE-PtrLBD39*.

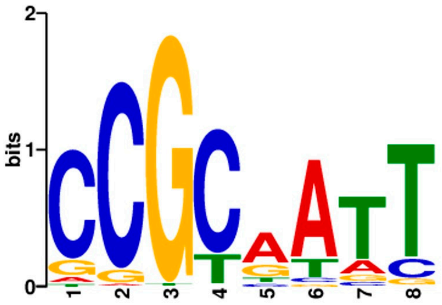

137 sites (79.2%), *P* = 1.8 × 10^{-16}

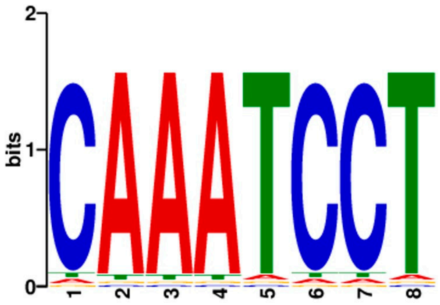

166 (96.0%) , *P* = 1.8 × 10^{-10}

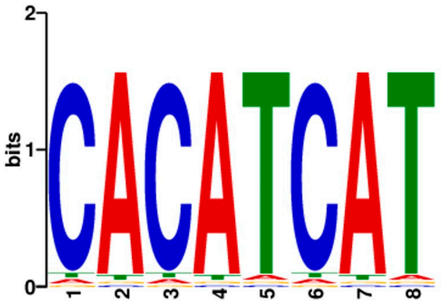

169 (97.7%) , *P* = 3.6 × 10^{-10}

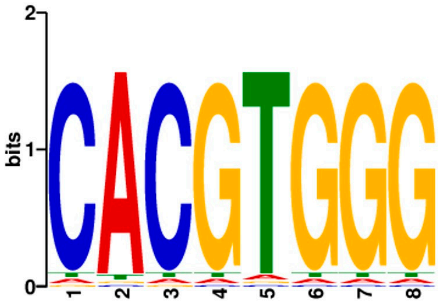

136 (78.6%) , *P* = 2.2 × 10^{-8}

Figure S5. Binding motifs within the peaks in promoters of PtrLBD39 target genes. The number of positive sequences matching the motif (percentage) and *P* value are listed under each motif.

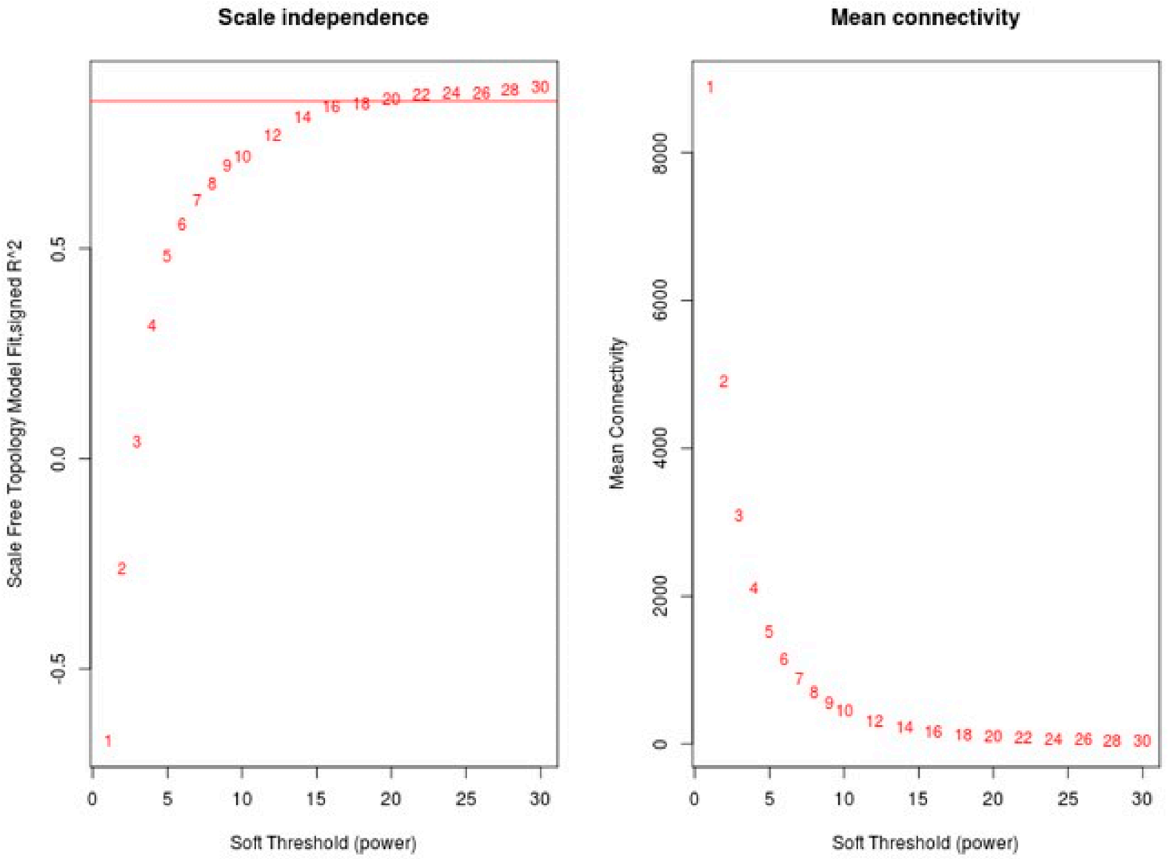

Figure S6. Soft threshold selection. The graph on the left: the horizontal axis represents the weight parameter β, the soft threshold, and the vertical axis represents the square of the log(k) and log(p(k)) correlation coefficients in the corresponding network. The graph on the right: the horizontal axis represents the weight parameter β, the soft threshold, and the vertical axis represents the mean of gene connectivity. According to the red line position in the left figure, the soft threshold selection is determined, that is, when $R^2 > 0.85$ and the platform is reached (the red line position is 0.85), the soft threshold is 20. Therefore, $\beta = 20$ was selected to build the network.

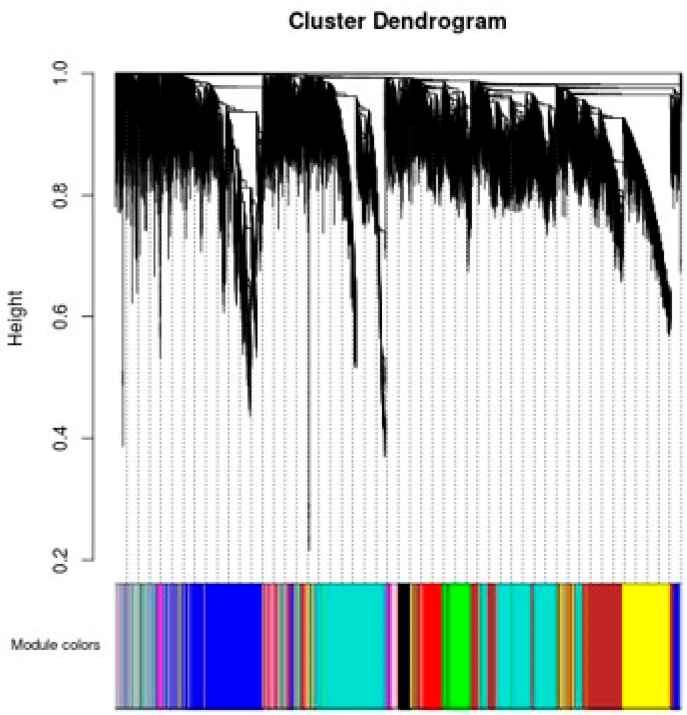

Figure S7. The network graph constructed by splitting modules using dynamic tree cutting algorithm. Each colored row represents a color-coded module which contains a group of highly connected genes. A total of 10 modules were identified. Grey modules represent genes that do not fit into any one module.

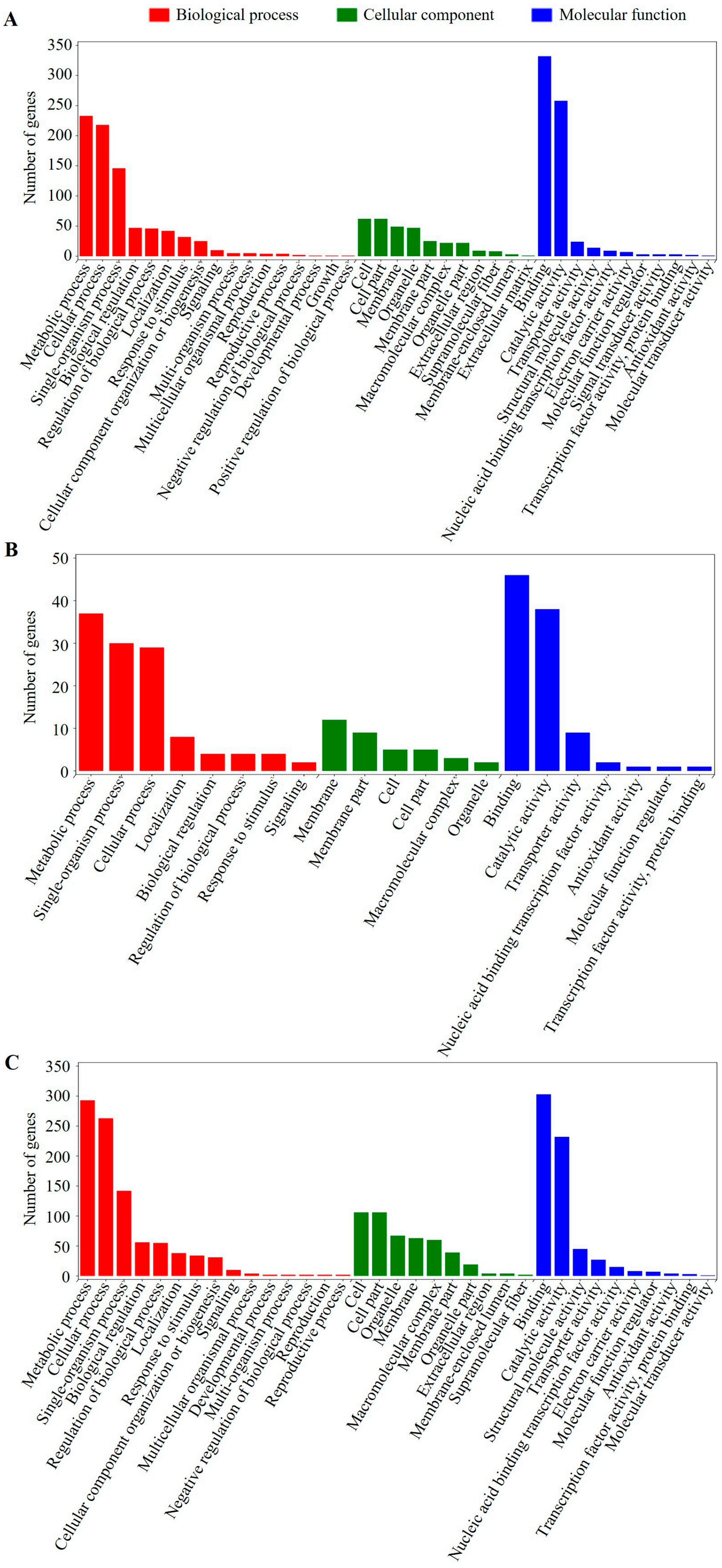
A
B
C
Biological process
Cellular component
Molecular function
Number of genes
Metabolic process
Cellular process
Single-organism process
Biological regulation
Regulation of biological process
Localization
Response to stimulus
Cellular component organization or biogenesis
Signaling
Multi-organism process
Multicellular organismal process
Reproduction
Reproductive process
Negative regulation of biological process
Developmental process
Growth
Positive regulation of biological process
Cell
Cell part
Membrane
Organelle
Membrane part
Macromolecular complex
Organelle part
Extracellular region
Supramolecular fiber
Membrane-enclosed lumen
Extracellular matrix
Binding
Catalytic activity
Transporter activity
Structural molecule activity
Nucleic acid binding transcription factor activity
Electron carrier activity
Molecular function regulator
Signal transducer activity
Transcription factor activity, protein binding
Antioxidant activity
Molecular transducer activity

Figure S8. GO enrichment analysis of the target modules. (**A**) yellow module. (**B**) black module. (**C**) brown module. The X-axis represents the GO functional classification, and the Y-axis represents the number of genes annotated on the GO entry.
