# Supplementary material for: Ectopic Expression of PtrLBD39 Retarded Primary and Secondary Growth in Populus trichocarpa
Source: Int J Mol Sci. 2024 Feb 12;25(4):2205. doi: 10.3390/ijms25042205 (PMC10889148; doi:10.3390/ijms25042205)
Supplement: Supplementary file 1 [file ijms-25-02205-s001.zip › Table S7.pdf]

Ectopic expression of *PtrLBD39* retarded primary and secondary growth in *Populus trichocarpa*

Supplementary Table

Table S7 Primer list

Gene name	Primer name	Primer sequence (5'-3')
PtrLBD39	*PtrLBD39*-F	CTAGTCTAGAATGAAGGAGAGCGGTCGA
PtrLBD39	*PtrLBD39*-R	CTAGGTCGACGCATGACCACAAAGACTC
PtrLBD39	RT-*PtrLBD39*-F	AGAGATGCAGTAAGTTCCATGGTT
PtrLBD39	RT-*PtrLBD39*-R	TCCTGTATTAGAGGAAGAAGTGA
PtrActin	RT-*PtrActin*-F	TGTTGCCCTTGACTATGAGCAGGA
PtrActin	RT-*PtrActin*-R	ACGGAATCTCTCAGCTCCAATGGT
